# Effects of plyometric training on physical fitness in adolescent and adult female team sport athletes: a systematic review and meta-analysis

**DOI:** 10.3389/fphys.2025.1639477

**Published:** 2025-09-18

**Authors:** Gesheng Lin, Ruli Zhang, Kai Wu, Beiwang Deng, Yuer Shi, Wenwei Huang, Jiaxin He, Jian Sun

**Affiliations:** ^1^ School of Athletic Training, Guangzhou Sport University, Guangzhou, Guangdong, China; ^2^ Guangdong Provincial Key Laboratory of Human Sports Performance Science, Guangzhou Sport University, Guangzhou, Guangdong, China; ^3^ Badminton Technical and Tactical Analysis and Diagnostic Laboratory, Guangzhou Sport University, Guangzhou, Guangdong, China

**Keywords:** plyometric exercise, athletic performance, training adaptations, female athletes, team sport

## Abstract

**Objective:**

This study aimed to investigate the effects of plyometric training (PT) on various physical fitness outcomes in adolescent and adult female team sport athletes.

**Methods:**

A systematic literature search was conducted across four electronic databases from inception to April 2025. Data analyses were performed using the meta and metafor packages in R version 4.4.3.

**Results:**

A total of 20 studies were included, involving 175 soccer players, 141 basketball players, 103 handball players, and 77 volleyball players. Compared with control groups, PT significantly improved countermovement jump (CMJ) height (ES: adolescents = 0.89; adults = 0.54) and change-of-direction (COD) performance (ES: adolescents = −1.42; adults = −0.86) in both adolescent and adult female athletes. However, significant improvement in 20-m sprint performance was observed only in adolescents (ES: adolescents = −0.99; adults = −0.32). Subgroup analyses showed that, compared to lower training accumulation, adolescents exhibited significantly greater improvements in CMJ height when the training duration was ≥9 weeks or the total sessions were ≥16. For COD performance, significantly greater improvements were observed when the training duration was ≥9 weeks, the total sessions were ≥18, and the total ground contacts were ≥1260.

**Conclusion:**

PT can significantly improve CMJ height and COD performance in both adolescent and adult female athletes, and significantly enhance sprint performance in adolescents. Compared to adults, adolescents demonstrate greater responsiveness to PT and are more sensitive to training accumulation.

**Systematic Review Registration:**

identifier CRD420251041817.

## 1 Introduction

Team sports such as basketball, volleyball, soccer, and handball demand a wide range of physical attributes from athletes to cope with the complex technical movements and high-intensity confrontations inherent in competitive play (Lidor and Ziv, 2010; Murr et al., 2018; Ortega-Becerra et al., 2020). During matches, players are frequently required to perform high-intensity actions such as jumping, sprinting, and rapid changes of direction, which are often critical determinants of game outcomes (Faude et al., 2012; Stojanović et al., 2018). These repeated explosive efforts place considerable physical demands on athletes, making physical fitness a fundamental component of success in team sports (Ramirez-Campillo et al., 2022). Moreover, superior physical fitness not only enhances technical execution and competitive performance but also reduces the risk of injury during intense physical contact (De la Motte et al., 2019).

Plyometric training (PT) has been widely recognized for its capacity to improve multiple components of physical performance (Markovic and Mikulic, 2010). Its effectiveness is primarily attributed to the stretch-shortening cycle (SSC)—a unique neuromuscular action involving consecutive eccentric, isometric, and concentric muscle contractions (Komi, 2003; Lloyd et al., 2011; Wilk et al., 1993). This mechanism efficiently utilizes stored elastic energy and stretch reflexes, thereby enhancing jump performance, sprint speed, and change-of-direction (COD) ability—all of which are critical for success in team sports (Asadi et al., 2017; Ramirez-Campillo et al., 2020a). Furthermore, a study by Kons et al. suggested that due to the jump-intensive nature of both training and competition in team sports, athletes may show higher responsiveness and greater training benefits from PT (Kons et al., 2023).

It is important to note that male and female athletes differ in several physiological and neuromuscular characteristics, including muscle fiber composition (with females having a greater proportion of type I fibers), muscle architecture (e.g., fascicle length and pennation angle), and SSC utilization capacity (only 64.1% in females compared to males) (Sáez-Sáez de Villarreal et al., 2010). Additionally, during growth and maturation, both neuromuscular recruitment and motor patterns evolve differently between sexes. For instance, males tend to demonstrate more pronounced improvements in strength, power, and coordination during maturation compared to females (Beunen and Malina, 1988; Kellis et al., 1999). While vertical jump height steadily increases with physical maturity in males, such improvements in females are less apparent (Beunen and Malina, 1988; Kellis et al., 1999). Therefore, given the biological differences (i.e., hormonal profile, menstrual cycle), it may therefore be erroneous to apply research conducted on male athletes to female athletes (Hughes et al., 2023).

In recent years, research on the effects of PT in female athletes has gradually increased. To date, six systematic reviews and meta-analyses (SRMA) have focused specifically on female populations (Cao et al., 2024; Chen et al., 2025; Moran et al., 2019; Ramirez-Campillo et al., 2020b; Sánchez et al., 2020; Stojanović et al., 2017). However, these reviews present several limitations: four of them failed to distinguish between adolescent and adult females due to small sample sizes (Cao et al., 2024; Ramirez-Campillo et al., 2020b; Sánchez et al., 2020; Stojanović et al., 2017), while the remaining two focused solely on adolescent females without addressing adult populations (Chen et al., 2025; Moran et al., 2019). Furthermore, half of the SRMAs assessed only vertical jump performance, neglecting other critical physical fitness outcomes such as sprinting and COD ability (Moran et al., 2019; Ramirez-Campillo et al., 2020b; Stojanović et al., 2017). Given the substantial physiological differences between adolescent and adult females—including maturity status, hormonal environment, neuromuscular development, and training responsiveness (Vescovi et al., 2011), this distinction is essential. Adolescents are typically in a developmental phase, and their adaptive responses to PT may differ considerably from those of physiologically mature adult females (Moran et al., 2019). Therefore, clearly differentiating between adolescent and adult female athletes when evaluating PT outcomes is critical for designing age-appropriate training strategies and optimizing practical applications.

Considering the multifaceted physical demands of female team sport athletes in competition, this study aims to provide a more comprehensive synthesis of evidence by examining the effects of PT on various physical fitness indicators in both adolescent and adult female team sport athletes.

## 2 Methods

This review was conducted according to the Preferred Reporting Items for Systematic Reviews and Meta-Analyses (PRISMA) guidelines for systematic reviews (Page et al., 2021). The study has been registered in the International Prospective Register of Systematic Reviews (PROSPERO: CRD420251041817).

### 2.1 Literature search: management and update

We conducted a systematic literature search across four electronic databases (PubMed, Web of Science, Scopus, and SPORTDiscus). Studies published from inception until April 2025 were included. The search strategy involved using Boolean operators AND and OR with the following keywords: “ballistic training”, “power training”, “plyometric*”, “stretch-shortening cycle”, “jump training”, “jump exercise*”, “women”, “girl*” and “female*”. The results of the systematic literature search from the four databases were combined and duplicates were removed. After the removal of duplicates, two researchers (GL and RZ) screened the search results based on the inclusion criteria. Any discrepancies between the two authors were resolved by consensus with a third author (KW). In addition, we screened the reference lists of both previous meta-analyses and the articles that met the inclusion and exclusion criteria. The detailed search strategies for each individual database are provided in Table 1.

**TABLE 1:** Detailed study retrieval strategies.

Database	Complete search strategy	Results
PubMed	((“ballistic training” [Title/Abstract] OR “power training” [Title/Abstract] OR “plyometric*” [Title/Abstract] OR “stretch-shortening cycle” [Title/Abstract] OR “jump training” [Title/Abstract] OR “jump exercise*” [Title/Abstract]) AND (women [Title/Abstract] OR girl*[Title/Abstract] OR female*[Title/Abstract]))	504
Web of Science	AB=(“ballistic training” OR “power training” OR “plyometric*” OR “stretch-shortening cycle” OR “jump training” OR “jump exercise*”) AND AB=(women OR girl* OR female*)	1,076
Scopus	TITLE-ABS-KEY (“ballistic training” OR “power training” OR “plyometric*” OR “stretch-shortening cycle” OR “jump training” OR “jump exercise*”) AND TITLE-ABS-KEY (women OR girl* OR female*)	1,814
SPORTDiscus	AB=(“ballistic training” OR “power training” OR “plyometric*” OR “stretch-shortening cycle” OR “jump training” OR “jump exercise*”) AND AB=(women OR girl* OR female*)	702

### 2.2 Inclusion and exclusion criteria

The studies were screened using the PICOS (Participants, Intervention, Comparators, Outcomes, and Study design) method (Liberati, 2009). Table 2 lists the inclusion/exclusion criteria. The additional inclusion criteria were as follows: (1) experimental trials published in peer-reviewed English-language journals.

**TABLE 2:** Eligibility criteria.

Category	Inclusion criteria	Exclusion criteria
Population	Female team-sport athletes	Male participants or non–team-sport athletes
Intervention	Plyometric training programs performed using bodyweight only and lasting for at least 4 weeks	Plyometric training performed with additional external resistance or integrated with resistance training
Comparator	Active control group (i.e., team sport athletes participating in regular training schedules)	Absence of active control group
Outcome	At least 1 measure of physical fitness (e.g., countermovement jump) before and after the training intervention	There are no indicators related to physical fitness (e.g., countermovement jump) before and after the training intervention
Study design	Randomized controlled trials	Non-randomized controlled trials

### 2.3 Data extraction

Microsoft Excel (Microsoft Corp., Redmond, WA, United States) was used to extract the means and standard deviations of the dependent variables before and after the intervention from the included studies. The first author (GL) extracted physical fitness indicators as dependent variables, along with participant characteristics (sample size, sport, and years of practice) and intervention details (frequency, duration, number of sessions, and total ground contacts). The second author (RZ) verified the accuracy and completeness of the extracted data. Any discrepancies between the two authors were resolved by consensus with a third author (KW). The age range for adolescents was defined according to the World Health Organization (10–19 years) (Organization, 2023), and the classification of adolescent and adult female athletes was based on the mean age reported in each included study.

### 2.4 Methodological quality and risk of bias

The Physiotherapy Evidence Database (PEDro) scale was used to assess the methodological quality of the included studies (Maher et al., 2003). The quality assessment was interpreted using the following 10-point scale: a score of ≤3 was considered to indicate poor quality, 4-5 indicated fair quality, and 6–10 indicated high quality. The PEDro scale consists of 11 items designed to evaluate methodological quality. Each satisfied item contributes 1 point to the overall PEDro score (range 0–10 points). Item 1 was not included in the quality rating of the studies as it pertains to external validity. The methodological quality of each included study was assessed independently by two authors (GL and RZ), and any discrepancies between the two authors were resolved via consensus with a third author (KW).

The risk of bias was assessed at the study level using the latest version of the Cochrane risk-of-bias tool for randomized trials (ROB2) (Flemyng et al., 2023) from five domains: randomization process, deviations from intended interventions, missing outcome data, measurement of the outcome, and selection of the reported result. Each item was rated as low risk, high risk, or some concerns. The quality of each included study was independently assessed by two reviewers (GL and RZ), and discrepancies were resolved by consultation with a third reviewer (KW).

### 2.5 Summary measures, synthesis of results, and publication bias

Data analyses were conducted using the meta and metafor packages in R version 4.4.3 (R Project for Statistical Computing). The pre- and post-training means and standard deviations for each dependent variable, including countermovement jump height, 20-m linear sprint performance, and change-of-direction performance, were used to calculate the effect sizes (ES; Hedge’s g) for each physical fitness indicator in both the PT and control groups. A random-effects model using the DerSimonian–Laird method was employed to account for variability between studies that might affect the PT effects (Deeks et al., 2019; Kontopantelis et al., 2013). ES values were expressed with 95% confidence intervals (95% CI). The calculated ES were interpreted using the following scale: trivial: <0.2; small: 0.2–0.6; moderate: >0.6–1.2; large: >1.2–2.0; very large: >2.0–4.0; and extremely large: >4.0 (Hopkins et al., 2009). Heterogeneity was assessed using the I^2^ statistic, with values of <25%, 25%–75%, and >75% representing low, moderate, and high levels of heterogeneity, respectively (Higgins and Thompson, 2002). The risk of publication bias was explored for continuous variables (≥10 studies per outcome) using the extended Egger’s test (Egger et al., 1997). In cases of bias, the trim and fill method was applied for adjustments (Duval and Tweedie, 2000). Statistical significance was set at p ≤ 0.05.

### 2.6 Subgroup analysis

Subgroup analyses were performed using median split techniques to divide moderator variables (frequency, training duration, total sessions and total ground contacts). The median was calculated when at least three studies provided data for the moderator variable.

### 2.7 Sensitivity analyses

We performed sensitivity analyses to assess the robustness of the summary estimates (e.g., p value, ES, I^2^). To examine the effects of each result from each study on the overall findings, results were analyzed with each study deleted from the model (automated leave-one-out analysis).

### 2.8 Certainty of evidence

Two authors (GL and RZ) rated the certainty of evidence (i.e., high; moderate; low; very low) using the Grading of Recommendations, Assessment, Development and Evaluation (GRADE) (Guyatt et al., 2011; Zhang et al., 2019a; Zhang et al., 2019b). The evidence started at a high level of certainty (per outcome), but was downgraded based on the following criteria: (i) Risk of bias in studies: judgments were downgraded by one level if the median PEDro scores were moderate (<6) or by two levels if they were poor (<4); (ii) Inconsistency: judgments were downgraded by one level when I^2^ was high (>75%); (iii) Indirectness: low risk of indirectness was attributed by default due to the specificity of populations, interventions, comparators and outcomes being guaranteed by the eligibility criteria; (iv) Imprecision: one level of downgrading occurred whenever < 800 participants were available for a comparison (Carr et al., 2008) and/or if there was no clear direction of the effects; When both were observed, certainty was downgraded by two levels. (v) Risk of publication bias: downgraded by one level if there was suspected publication bias.

## 3 Results

### 3.1 Study selection

**FIGURE 1:**
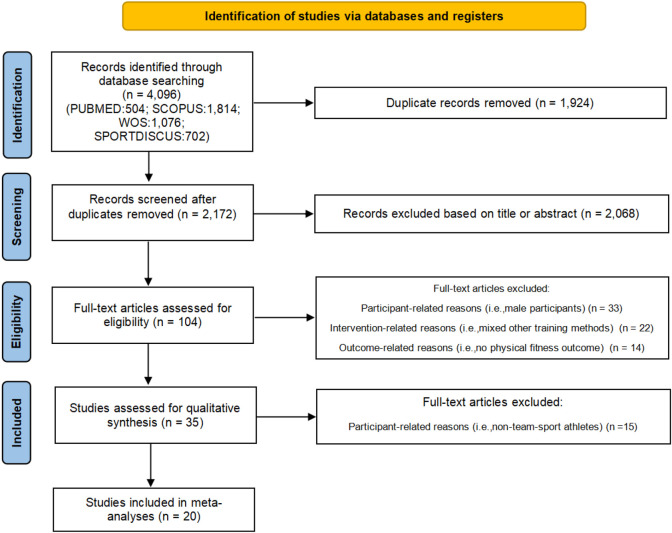
Study inclusion and exclusion selection process.

The search process identified 4096 studies (504 from PubMed, 1814 from Scopus, 1076 from Web of Science, and 702 from SPORTDiscus), resulting in a total of 2172 studies after removing duplicates. Ultimately, 20 studies were included in this meta-analysis. Figure 1 illustrates the study selection process. Table 3 displays the characteristics of participants in the included studies.

**TABLE 3:** Characteristics of participants examined in the included studies.

Study	n^a^	Age^b^ (years)	Height^b^ (cm)	Body mass^b^ (kg)	Sport	Frequency (days/week)	Duration (weeks)	Total ground contacts
Adolescents								
Attene et al. (2015)	18/18	14.83/15.2	163/165	51.89/57.5	Basketball	2	6	1120
Gaamouri et al. (2023)	14/14	15.7/15.8	165/167	63.8/63.3	Handball	2	10	1440
Haghighi et al. (2024)	8/8	14.6/15.1	168.3/165.8	61.7/56.7	Basketball	2	6	1188
Hammami et al. (2020)	17/17	15.8/15.8	166/167	64.2/63	Handball	2	10	1440
Hammami et al. (2019)	21/20	13.5/13.3	142/143	42.6/42.3	Handball	2	9	1260
Idrizovic et al. (2018)	13/17	16.6/16.6	174.1/175.9	62.7/59.4	Volleyball	2	12	1226
Meszler and Váczi. (2019)	9/9	15.8/15.7	176.4/177.5	63.5/66.1	Basketball	2	7	1027
Ozbar et al. (2014)	9/9	18.3/18	163.1/159.4	58.8/54.4	Soccer	1	8	1210
Paes et al. (2022)	11/10	14.45/15.3	160/163	53.72/59.93	Basketball	2	6	900
Pereira et al. (2015)	10/10	14/13.8	160/160	52/53.5	Volleyball	2	8	2376
Rojano Ortega et al. (2022)	14/13	16.07/15.71	166.79/165.57	67.82/61.3	Volleyball	2	7	1612
Adults								
Cherni et al. (2020)	15/12	20.9/21	172/173	65.1/67.3	Basketball	2	8	1584
Fischetti et al. (2019)	14/14	26.5/26.7	160.1/160.7	60.8/60.6	Soccer	2	12	3240
Maciejczyk et al. (2021)	7/8	21/18.2	164.5/161.7	61.3/55	Soccer	2	4	524
Nonnato et al. (2022)	8/8	23/23	167/167	60.3/60.3	Soccer	1	12	1488
Ramirez-Campillo et al. (2018)	8/8/7	22.8/21.4/20.1	158/157.6/160.1	54.9/59.6/55.3	Soccer	1/2	8	1020
Ramírez-Campillo et al. (2016)	19/19	22.4/20.5	159/161	60.7/60.2	Soccer	2	6	2400
Rosas et al. (2017)	8/9	22.8/24	164/162	61.1/58.5	Soccer	2	6	2400
Sánchez-Sixto et al. (2021)	11/12	22.55/22.58	166/169	64.05/65.77	Basketball	2	6	512
Sedano Campo et al. (2009)	10/10	22.8/23	163/161.5	58.5/56.9	Soccer	3	12	3240

^a^ The sample size of the experimental group/control group.

^b^ The mean values of the experimental group/control group.

### 3.2 Methodological quality and risk of bias assessment

Using the PEDro checklist, 4 studies were considered to be of moderate quality (4-5 points), and the remaining 16 studies were considered to be of high quality (6–10 points). The results of the methodological quality assessment are presented in Table 4.

**TABLE 4:** Rating of studies according to the Physiotherapy Evidence Database (PEDRo) scale.

Study	1	2	3	4	5	6	7	8	9	10	11	Score	Study quality
Attene et al. (2015)	1	1	0	1	0	0	0	1	1	1	1	6	High
Cherni et al. (2020)	1	1	0	1	0	0	0	1	1	1	1	6	High
Fischetti et al. (2019)	1	1	0	1	0	0	1	1	1	1	1	7	High
Gaamouri et al. (2023)	1	1	0	1	0	0	0	1	1	1	1	6	High
Haghighi et al. (2024)	1	1	1	1	0	0	0	1	1	1	1	7	High
Hammami et al. (2020)	1	1	0	1	0	0	0	1	1	1	1	6	High
Hammami et al. (2019)	1	1	0	1	0	0	0	1	1	1	1	6	High
Idrizovic et al. (2018)	1	1	0	1	0	0	0	1	0	1	1	5	Moderate
Maciejczyk et al. (2021)	1	1	0	1	0	0	0	1	0	1	1	5	Moderate
Meszler and Váczi (2019)	1	1	0	1	0	0	0	1	1	1	1	6	High
Nonnato et al. (2022)	1	1	0	1	0	0	0	1	1	1	1	6	High
Ozbar et al. (2014)	1	1	0	1	0	0	0	1	1	1	1	6	High
Paes et al. (2022)	1	1	0	1	0	0	0	1	0	1	1	5	Moderate
Pereira et al. (2015)	1	1	0	1	0	0	0	1	1	1	1	6	High
Ramirez-Campillo et al. (2018)	1	1	0	1	0	0	1	0	0	1	1	5	Moderate
Ramírez-Campillo et al. (2016)	1	1	0	1	0	0	1	1	0	1	1	6	High
Rojano Ortega et al. (2022)	1	1	0	1	0	0	0	1	1	1	1	6	High
Rosas et al. (2017)	1	1	0	1	1	0	1	1	1	1	1	8	High
Sánchez-Sixto et al. (2021)	1	1	0	1	0	0	0	1	1	1	1	6	High
Sedano Campo et al. (2009)	1	1	0	1	0	0	0	1	1	1	1	6	High

**FIGURE 2:**
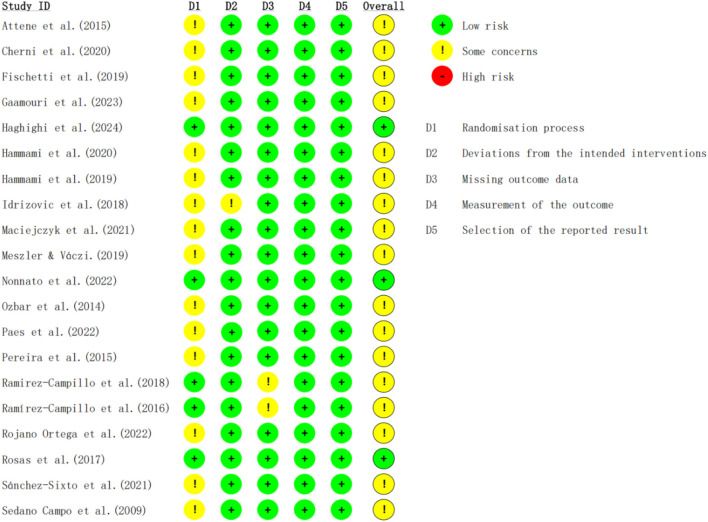
Risk of bias for each study.

**FIGURE 3:**
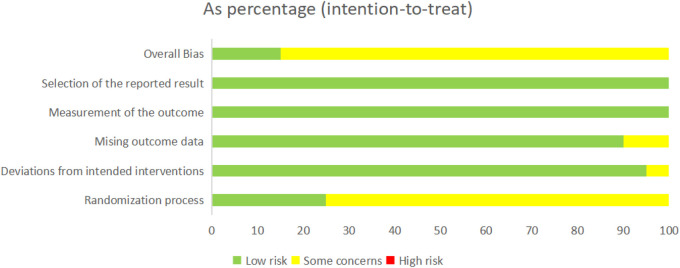
Risk of overall bias.

Additionally, to provide a more comprehensive assessment of bias risk, we employed the Cochrane ROB2 tool to evaluate each domain of bias in the included studies systematically. The results of the ROB2 assessment are shown in Figure 2 and Figure 3. Overall, three studies were classified as having a low risk of bias, while the remaining seventeen were classified as raising some concerns. Regarding the randomization process, only five studies reported allocation concealment, with the others providing no relevant information; therefore, most studies in this domain were rated as “some concerns.” In terms of deviations from intended interventions, one study was rated as “some concerns” due to intervention adjustments. For missing outcome data, two studies were rated as “some concerns” because they excluded participants with a low completion rate. In other domains, all studies were rated as low risk.

### 3.3 Results of the meta-analysis

**FIGURE 4:**
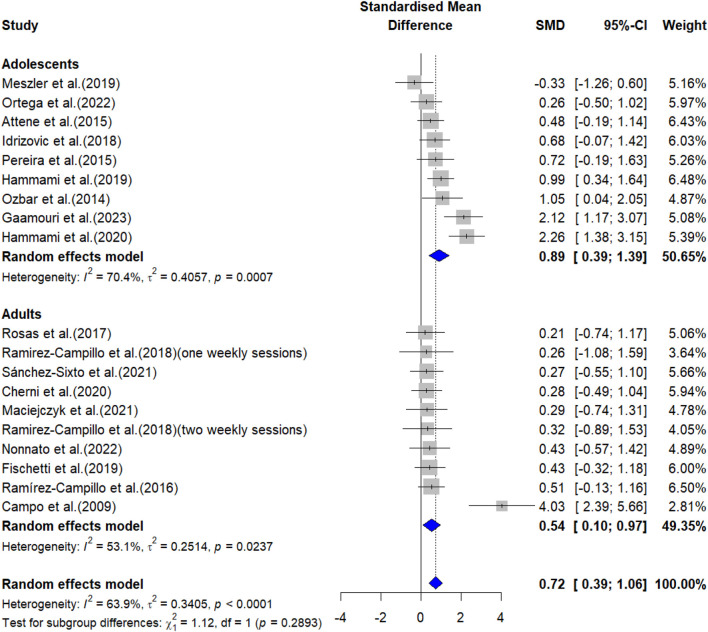
Forest plot showing the effects of plyometric training on countermovement jump performance in female team-sport athletes.

**FIGURE 5:**
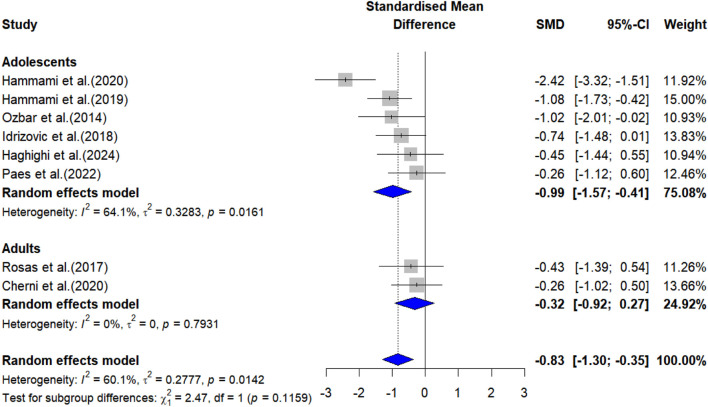
Forest plot showing the effects of plyometric training on 20-m linear sprint performance in female team-sport athletes.

**FIGURE 6:**
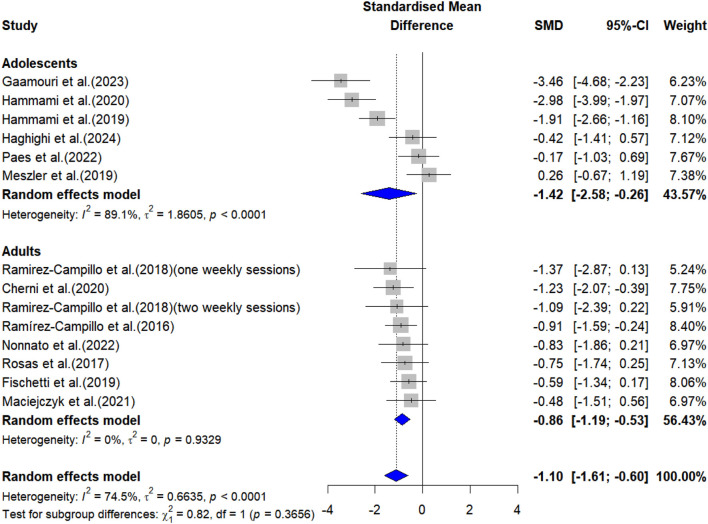
Forest plot showing the effects of plyometric training on change-of-direction performance in female team-sport athletes.

The overall effects of PT on physical fitness are shown in Table 5. The forest plots are shown in Figures 4–6.

**TABLE 5:** Results of the meta-analysis.

	n^a^	ES (95%CI)	%Weight	p	I^2^	Egger’s test (p)
Countermovement jump height						
Adolescents	9	0.89 (0.39–1.39)	50.65%	<0.001	70.4%	-
Adults	10	0.54 (0.10–0.97)	49.35%	0.017	53.1%	0.132
All	19	0.72 (0.39–1.06)	100%	<0.001	63.9%	0.213
20-m linear sprint performance						
Adolescents	6	−0.99 (-1.57 to −0.41)	75.08%	<0.001	64.1%	-
Adults	2	−0.32 (-0.92 to 0.27)	24.92%	0.289	0%	-
All	8	−0.83 (-1.30 to −0.35)	100%	<0.001	60.1%	-
Change-of-direction performance						
Adolescents	6	−1.42 (-2.58 to −0.26)	43.57%	0.017	89.1%	-
Adults	8	−0.86 (-1.19 to −0.53)	56.43%	<0.001	0%	-
All	14	−1.10 (-1.61 to −0.60)	100%	<0.001	74.5%	0.481

^a^ The data are represented as the number of studies providing data.

Abbreviations: ES, effect sizes (Hedge’s g); 95%CI, 95% confidence interval; All = Youth + Adolescents.

#### 3.3.1 Jump performance

Meta-analysis results indicated that PT had a significant effect on CMJ height in both adolescent [ES = 0.89, 95% CI: (0.39, 1.39), P < 0.001, I^2^ = 70.4%] and adult [ES = 0.54, 95% CI: (0.10, 0.97), P = 0.017, I^2^ = 53.1%] female team sport athletes. After the sensitivity analyses (automated leave-one-out analysis), the robustness of the summary estimates (e.g., p-value, ES) for both adolescents and adults was confirmed.

#### 3.3.2 Linear sprint performance

Meta-analysis results indicated that PT significantly improved 20-m sprint performance in adolescent female athletes [ES = −0.99, 95% CI: (−1.57, −0.41), P < 0.001, I^2^ = 64.1%], whereas no significant effect was observed in adult female athletes [ES = −0.32, 95% CI: (−0.92, 0.27), P = 0.289, I^2^ = 0%]. After the sensitivity analyses (automated leave-one-out analysis), the robustness of the summary estimates (e.g., p-value, ES) for both adolescents and adults was confirmed.

#### 3.3.3 Change-of-direction performance

Meta-analysis results indicated that PT had a significant effect on COD performance in both adolescent [ES = −1.42, 95% CI: (−2.58, −0.26), P = 0.017, I^2^ = 89.1%] and adult [ES = −0.86, 95% CI: (−1.19, −0.53), P < 0.001, I^2^ = 0%] female team sport athletes. After the sensitivity analyses (automated leave-one-out analysis), the robustness of the summary estimates (e.g., p-value, ES) for adults was confirmed. For adolescents, the results lost statistical significance (all P > 0.05) when any one of the studies by Gaamouri et al. (2023), or Hammami et al. (2019); 2020) was excluded.

### 3.4 Results of subgroup analysis

The results of the subgroup analyses are presented in Table 6.

**TABLE 6:** Results of subgroup analysis.

		n^a^	ES (95%CI)	%Weight	I^2^	Between group p
Countermovement jump height (adolescents)						
Duration	≥9 weeks	5	1.46 (0.70–2.22)	45.27%	72.5%	0.017
	≤8 weeks	4	0.42 (0.02–0.82)	54.73%	13.6%	
Total Sessions	≥16 sessions	5	1.32 (0.68–1.96)	55.73%	67.8%	0.018
	≤14 sessions	4	0.35 (-0.13–0.83)	44.27%	27%	
Total ground contacts	≥1260 contacts	5	1.24 (0.50–1.99)	55.62%	75.7%	0.091
	≤1226 contacts	4	0.47 (-0.02–0.97)	44.38%	32.1%	
Countermovement jump height (Adults)						
Frequency	≥2 sessions/week	8	0.59 (0.06–1.13)	83.22%	63.3%	0.639
	1 sessions/week	2	0.37 (-0.43–1.16)	16.78%	0%	
Duration	≥8 weeks	6	0.79 (-0.02–1.61)	54.79%	72.6%	0.353
	≤7 weeks	4	0.36 (-0.05–0.77)	45.21%	0%	
Total Sessions	≥16 sessions	4	1.08 (-0.15–2.31)	38.02%	83.2%	0.271
	≤12 sessions	6	0.36 (-0.00–0.73)	61.98%	0%	
Total ground contacts	≥1584 contacts	5	0.85 (0.01–1.68)	54.15%	78.2%	0.273
	≤1488 contacts	5	0.31 (-0.15–0.78)	45.85%	0%	
20-m linear sprint performance (Adolescents)						
Duration/Total sessions/Total ground contacts	≥9 weeks/≥18 sessions/1226 contacts	3	−1.37 (-2.28 to −0.46)	53.85%	76.3%	0.126
	≤8 weeks/≤12 sessions/1210 contacts	3	−0.54 (-1.09 to 0.00)	46.15%	0%	
Change-of-direction performance (Adolescents)						
Duration/Total sessions/Total ground contacts	≥9 weeks/≥18 sessions/1260 contacts	3	−2.69 (-3.64 to −1.75)	49.55%	64.1%	<0.001
	≤7 weeks/≤14 sessions/1188 contacts	3	−0.10 (-0.63 to 0.43)	50.45%	0%	
Change-of-direction performance (Adults)						
Frequency	≥2 sessions/week	6	−0.84 (-1.19 to −0.48)	85.15%	0%	0.73
	1 sessions/week	2	−1.00 (-1.85 to −0.15)	14.85%	0%	
Duration	≥8 weeks	5	−0.93 (-1.38 to −0.49)	55.16%	0%	0.635
	≤7 weeks	3	−0.77 (-1.26 to −0.28)	44.84%	0%	
Total Sessions	≥16 sessions	3	−0.91 (-1.43 to −0.39)	40.31%	0%	0.818
	≤14 sessions	5	−0.83 (-1.26 to −0.41)	59.69%	0%	
Total ground contacts	≥1584 contacts	4	−0.87 (-1.26 to −0.47)	68.79%	0%	0.956
	≤1488 contacts	4	−0.85 (-1.44 to −0.26)	31.21%	0%	

^a^ The data are represented as the number of studies providing data.

Abbreviations: ES, effect sizes (Hedge’s g); 95%CI, 95% confidence interval.

For improvements in CMJ height among adolescent athletes, significantly greater gains were observed with ≥9 weeks of PT compared to ≤8 weeks [ES = 1.46 vs. 0.42, P = 0.017]. Likewise, PT programs involving ≥16 total sessions produced more substantial improvements than those with ≤14 sessions [ES = 1.32 vs. 0.35, P = 0.018].

For improvements in COD performance among adolescent athletes, significantly greater gains were observed in subgroup analyses when PT lasted ≥9 weeks, included ≥18 total sessions, or involved ≥1260 total ground contacts, compared to training durations of ≤7 weeks, ≤14 sessions, or ≤1188 contacts, respectively [ES = −2.69 vs. −0.10, P < 0.001]. These three comparisons were based on the same dataset.

### 3.5 Certainty of evidence

According to the GRADE assessment (Table 7), the certainty of evidence was considered moderate to low for the main analyses.

**TABLE 7:** GRADE analyses.

Participants (studies)	Risk of bias	Inconsistency	Indirectness	Imprecision	Publication bias	Certainty of evidence
Countermovement jump height (Adolescents)						
127 (9)	No downgrading	No downgrading	No downgrading	Downgraded by one level	No downgrading	⨁⨁⨁◯ Moderate
Countermovement jump height (Adults)						
207 (10)	No downgrading	No downgrading	No downgrading	Downgraded by one level	No downgrading	⨁⨁⨁◯ Moderate
20-m linear sprint performance (Adolescents)						
160 (6)	No downgrading	No downgrading	No downgrading	Downgraded by one level	No downgrading	⨁⨁⨁◯ Moderate
20-m linear sprint performance (Adults)						
44 (2)	No downgrading	No downgrading	No downgrading	Downgraded by two levels	No downgrading	⨁⨁◯◯ Low
Change-of-direction performance (Adolescents)						
158 (6)	No downgrading	Downgraded by one level	No downgrading	Downgraded by one level	No downgrading	⨁⨁◯◯ Low
Change-of-direction performance (Adults)						
164 (8)	No downgrading	No downgrading	No downgrading	Downgraded by one level	No downgrading	⨁⨁⨁◯ Moderate

## 4 Discussion

### 4.1 Jump performance

The meta-analysis indicated that PT significantly improved CMJ height in both adolescent and adult female athletes, with adolescents demonstrating greater gains, although the between-group difference was not statistically significant. Subgroup analyses further revealed that adolescent athletes experienced significantly greater improvements in jump performance when the training duration was ≥9 weeks or the total number of sessions was ≥16, compared to those with lower training accumulation.

The improvement in CMJ height may result from various neuromuscular adaptations, including enhanced neural drive of the agonist muscles, changes in the stiffness of the muscle–tendon unit, improvements in muscle architecture (e.g., increased muscle fiber cross-sectional area and fascicle length), better intermuscular coordination, and heightened stretch reflex excitability (Markovic and Mikulic, 2010). According to the principle of training specificity, the typical explosive movements involved in PT reinforce neuromuscular control patterns similar to those used in CMJ, particularly during the eccentric–concentric transition of the SSC. This repeated stimulation may enhance the efficiency of elastic energy storage and release, thereby facilitating improvements in jump performance (Stojanović et al., 2017).

Compared to adult females, adolescent female athletes exhibited greater improvements in jump performance following PT, which may be attributed to the synergistic effect of training adaptation and natural developmental processes. Lloyd et al. noted that the neuromuscular system during adolescence has not yet reached the stable state of adults and is in a phase of dynamic maturation (Lloyd et al., 2011). On one hand, structural components such as tendon stiffness and joint stiffness in adolescents gradually optimize with age; meanwhile, neuromodulatory capabilities including motor unit recruitment efficiency, muscle preactivation levels, and stretch reflex responses also show age-related improvements (Radnor et al., 2018). This natural development can promote the enhancement of SSC function even without specialized training (Malina et al., 2004; Radnor et al., 2018). On the other hand, the muscle activation strategy of adolescents is not yet fixed and is still in the transition stage from “reactive protective inhibition” to “performance-enhancing excitation” (Lambertz et al., 2003). Compared with adults with stable neuromuscular function, their systems are more sensitive to plyometric training stimuli. Training can more easily enhance the elastic energy utilization efficiency and reflexive muscle activation effect in SSC (Lloyd et al., 2011). This synergy between “the basic improvement of SSC brought by natural development” and “the neuromuscular adaptive improvements induced by training” amplifies the promoting effect of PT on SSC efficiency (Lloyd et al., 2011). In contrast, the neuromuscular function of adult females has reached a mature and stable state, lacking such a synergistic advantage, thus limiting the magnitude of improvement.

In addition, training variable analyses revealed that longer training duration and a higher number of sessions significantly enhanced CMJ performance among adolescents, while no significant differences were observed across training variables in adult females. This may reflect a heightened sensitivity of the adolescent neuromuscular system during a critical window of developmental plasticity. In contrast, adult athletes typically exhibit higher baseline jump performance, and their adaptation potential may be closer to saturation, thereby limiting the influence of training variable differences on performance outcomes (Stojanović et al., 2017).

### 4.2 Sprint performance

The meta-analysis results showed that PT significantly improved 20-m sprint performance in adolescent female athletes, whereas no statistically significant improvements were observed in adult females.

The sprint-enhancing effects of PT in adolescents may be attributed to a combination of physiological and neuromuscular mechanisms. First, PT enhances lower-limb maximal strength and explosive power, which contributes to increased stride length and horizontal force output during the acceleration phase (Christou et al., 2006; Markovic and Mikulic, 2010). Second, plyometric exercises often involve high-frequency explosive jumps and rapid eccentric-to-concentric transitions, which exhibit strong movement specificity to sprinting. These characteristics help reduce ground contact time, thereby improving step frequency and overall sprint efficiency (Rimmer and Sleivert, 2000). From a neural perspective, PT may increase motor unit recruitment, enhance joint proprioception, and improve neuromuscular control, which facilitates better coordination of movement rhythm and posture during sprint acceleration (Asadi, 2013a). These mechanisms may work synergistically to enhance sprint performance, particularly in the initiation and acceleration phases.

In contrast, adult females did not show significant improvements in 20-m sprint performance following PT intervention. Among the two studies included, Cherni et al. reported a non-significant trend of improvement in the PT group (Cherni et al., 2020). The authors suggested that the plyometric exercises used in their protocol involved relatively long ground contact times, which may not effectively replicate the neuromuscular demands of short contact times required during sprinting, thus limiting the transferability of training adaptations (Cherni et al., 2020). Beyond the limitations of training design, individual developmental stage may also influence the magnitude of training adaptations. Adolescents are in a period of rapid growth, and the ongoing development of limb length and skeletal structure naturally promotes longer stride length and improved sprint efficiency (Asadi et al., 2018; Silva et al., 2022). In contrast, adult females have completed physical maturation, and their sprint performance may have a lower ceiling for adaptation compared to adolescents. Given the limited number of studies involving adult females, further research is needed to explore the responsiveness of this population to PT interventions targeting sprint performance.

Further analysis of training variables in adolescents revealed a trend toward greater improvement with higher levels of training accumulation, although between-group differences were not statistically significant. Zhou et al. found that, among adolescent basketball players, total jump count was strongly correlated with both sprint and COD performance (Zhou et al., 2024). This may be explained by the fact that higher repetition volumes result in more frequent activation of joint mechanoreceptors, thereby enhancing proprioception and motor control, ultimately improving sprint acceleration performance (Asadi, 2013b).

### 4.3 Change-of-direction performance

The meta-analysis revealed that PT significantly improved COD performance in both adolescent and adult female athletes, with greater improvements observed in adolescents, although the between-group difference was not statistically significant. Subgroup analyses further showed that significantly greater improvements in adolescents occurred when the training duration was ≥9 weeks, the total number of sessions was ≥18, and the total number of ground contacts was ≥1260, compared to those with lower training accumulation.

The improvements in COD ability following PT may primarily be attributed to neuromuscular adaptations, including increased motor unit recruitment and firing frequency (Aagaard et al., 2002; Radnor et al., 2018). Specifically, COD performance depends on rapid force development, eccentric control of the thigh musculature, and efficient coordination of the lower-limb extensors during the eccentric-to-concentric transition. PT has been shown to enhance these key capabilities (Aagaard et al., 2002; Miller et al., 2006). These neuromuscular adaptations not only increase lower-limb force output but also optimize rhythm and postural control during high-speed directional changes, thereby improving overall COD efficiency.

Compared to adult females, adolescent female athletes demonstrated greater improvements in COD performance following PT, which is consistent with the improvements observed in their CMJ performance. This similar pattern supports our hypothesis that the greater improvements seen in adolescents may be attributed to a synergistic interaction between training-induced adaptations and natural maturation. COD performance may be more influenced by motor control factors, such as skill execution and coordination, rather than by strength or power alone (Young et al., 2002). Adolescents tend to exhibit greater plasticity in intermuscular coordination, stretch reflex excitability, SSC utilization, and neural drive to the prime movers (Markovic and Mikulic, 2010), which may explain their enhanced gains in COD performance following PT.

It is important to note that although the meta-analysis showed a significant improvement in COD performance among adolescent athletes following PT, a high level of heterogeneity was observed (I^2^ = 89.1%). Sensitivity analysis further revealed that the statistical significance disappeared when either the study by Gaamouri et al. or Hammami et al. was excluded. Notably, as shown by the subgroup analysis, these three studies comprised the entire high training accumulation group (training duration ≥9 weeks, ≥18 total sessions, and ≥1260 total ground contacts), and this subgroup exhibited a more pronounced improvement in COD (ES = −2.69 vs. −0.10). This finding implies that the significant improvements observed in adolescents may have been largely attributable to studies involving higher training accumulation. Since improvements in COD performance rely more heavily on neuromuscular adaptations, insufficient training volume may not generate adequate muscle engagement or neural activation (Ramírez-Campillo et al., 2013). Therefore, we recommend appropriately increasing training accumulation while ensuring a balance between training load and recovery, in order to enhance PT adaptations in adolescent athletes. Future research should further investigate the dose–response relationship between training volume and neuromuscular adaptations to develop more targeted intervention strategies.

### 4.4 Limitations

A key limitation of this systematic review and meta-analysis is the insufficient methodological consideration for female athletes. As Elliott-Sale et al. noted, previous studies involving female participants have often failed to account for the menstrual cycle in their methodologies, which further complicates the derivation of evidence-based recommendations (Elliott-Sale et al., 2021). Research has shown that fluctuations in estrogen levels during the menstrual cycle may influence central nervous system fatigue, tendon and ligament strength, and muscle function, thereby leading to decreased sports performance or impaired adaptive responses to training (Emmonds et al., 2019). In the present study, one included study reported that all participants had regular menstrual cycles (Cherni et al., 2020), while another explicitly acknowledged that the menstrual cycle was not considered in its research process (Gaamouri et al., 2023). For the remaining studies, it was impossible to determine whether necessary methodological considerations (e.g., controlling for menstrual cycle phases) were implemented to address the known physiological differences between sexes. Therefore, we encourage future similar studies to explicitly incorporate menstrual cycle monitoring or control to reduce the interference of hormonal fluctuations on research results.

Additionally, athletes’ maturation stage may also modulate the effects of PT, a factor that could not be fully explored in this study. Existing research indicates that the responses of male adolescent athletes to PT vary by maturation stage (Asadi et al., 2017; Moran et al., 2017). However, due to the limited sample size of female adolescent athletes included in this study, we were unable to further stratify different maturation stages to analyze their differential effects on PT outcomes. Thus, we encourage future studies to further investigate the impact of different maturation stages on the adaptive mechanisms of PT in female adolescent athletes.

## 5 Practical applications

Based on the current findings, PT significantly improves CMJ and COD performance in adolescent and adult female team-sport athletes, whereas significant improvements in 20 m sprint performance were observed only in adolescents; compared with adults, adolescents show greater overall responsiveness to PT and are more sensitive to training accumulation. In practice, we recommend appropriately increasing training accumulation for adolescents while ensuring a balance between training and recovery and maintaining movement quality. It should be noted that COD results in adolescents showed high heterogeneity; if training accumulation is insufficient, the practical gains may be limited. Overall, we recommend a PT program delivered twice per week over ≥9 weeks, with ≥18 total sessions and ≥1260 total ground contacts, as this was associated with more pronounced improvements in CMJ and COD performance. Adult athletes can employ PT to improve jumping and COD performance; however, when the goal is to enhance sprint ability, we suggest selecting exercises characterized by relatively short ground-contact times, as these better match the neuromuscular demands of sprint performance (Cherni et al., 2020).

## 6 Conclusion

Compared to control groups, PT significantly improved CMJ height and COD performance in both adolescent and adult female athletes, while significant improvements in 20-m sprint performance were observed only in adolescents. Adolescent athletes showed greater responsiveness to PT, which may be attributed to the combined effects of training adaptations and natural maturation. They also appeared more sensitive to training accumulation. Specifically, greater improvements in CMJ were observed when training duration was ≥9 weeks or total sessions were ≥16. For COD, greater improvements occurred when training duration was ≥9 weeks, total sessions were ≥18, and total ground contacts were ≥1260. Therefore, we recommend appropriately increasing training accumulation, while ensuring a balance between training load and recovery, to enhance PT adaptations in adolescent athletes.

## Data Availability

The original contributions presented in the study are included in the article/Supplementary Material, further inquiries can be directed to the corresponding authors.

## Abbreviations

PT, Plyometric training; SSC, Stretch–shortening cycle; CMJ, Countermovement jump; COD, Change-of-direction; SRMA, Systematic Review and Meta-Analysis; PEDro, Physiotherapy Evidence Database; CI, Confidence interval; ES, Effect size; I^2^, Impact of statistical heterogeneity; PICOS, Participants, intervention, comparators, outcomes, and study design.

## References

[B1] Aagaard P. Simonsen E. B. Andersen J. L. Magnusson P. Dyhre-Poulsen P. (2002). Increased rate of force development and neural drive of human skeletal muscle following resistance training. J. Appl. Physiology 93 (4), 1318–1326. 10.1152/japplphysiol.00283.2002 12235031

[B2] Asadi A. (2013a). Effects of in-season plyometric training on sprint and balance performance in basketball players. Sport Sci. 6 (1), 24–27.

[B3] Asadi A. (2013b). Effects of in-season short-term plyometric training on jumping and agility performance of basketball players. Sport Sci. Health 9 (3), 133–137. 10.1007/s11332-013-0159-4

[B4] Asadi A. Arazi H. Ramirez-Campillo R. Moran J. Izquierdo M. (2017). Influence of maturation stage on agility performance gains after plyometric training: a systematic review and meta-analysis. J. Strength Cond. Res. 31 (9), 2609–2617. 10.1519/JSC.0000000000001994 28557853

[B5] Asadi A. Ramirez-Campillo R. Arazi H. Sáez de Villarreal E. (2018). The effects of maturation on jumping ability and sprint adaptations to plyometric training in youth soccer players. J. Sports Sci. 36 (21), 2405–2411. 10.1080/02640414.2018.1459151 29611771

[B6] Attene G. Iuliano E. Di Cagno A. Calcagno G. Moalla W. Aquino G. (2015). Improving neuromuscular performance in young basketball players: plyometric vs. technique training. J. Sports Med. Phys. Fit. 55 (1–2), 1–8. 24921611

[B7] Beunen G. Malina R. M. (1988). Growth and physical performance relative to the timing of the adolescent spurt. Exerc. Sport Sci. Rev. 16, 503–540. 10.1249/00003677-198800160-00018 3292266

[B8] Cao S. Wang Z. Guo J. Geok S. K. Sun H. Liu J. (2024). The effects of plyometric training on physical fitness and skill-related performance in female basketball players: a systematic review and meta-analysis. Front. Physiology 15, 1386788. 10.3389/fphys.2024.1386788 39027901 PMC11254773

[B9] Carr A. Dawson B. Schneiker K. Goodman C. Lay B. (2008). Effect of caffeine supplementation on repeated sprint running performance. J. SPORTS Med. Phys. Fit. 48 (4), 472–478. 18997650

[B10] Chen L. Qu W. Yan R. Deng B. Sun J. Wang Y. (2025). Timing is everything: the age-related impact of plyometric training on lower limb explosive strength in Male adolescents and its general effectiveness in female adolescents. Eur. J. Appl. Physiology 125, 1665–1685. 10.1007/s00421-024-05683-0 39751817

[B11] Cherni Y. Hammami M. Jelid M. C. Aloui G. Suzuki K. Shephard R. J. (2020). Neuromuscular adaptations and enhancement of physical performance in female basketball players after 8 weeks of plyometric training. Front. Physiology 11, 588787. 10.3389/fphys.2020.588787 33584327 PMC7873906

[B12] Christou M. Smilios I. Sotiropoulos K. Volaklis K. Pilianidis T. Tokmakidis S. P. (2006). Effects of resistance training on the physical capacities of adolescent soccer players. J. Strength and Cond. Res. 20 (4), 783–791. 10.1519/R-17254.1 17194231

[B13] de la Motte S. J. Lisman P. Gribbin T. C. Murphy K. Deuster P. A. (2019). Systematic review of the association between physical fitness and musculoskeletal injury risk: Part 3-flexibility, power, speed, balance, and agility. J. Strength and Cond. Res. 33 (6), 1723–1735. 10.1519/JSC.0000000000002382 29239989

[B14] Deeks J. J. Higgins J. P. Altman D. G. on behalf of the Cochrane Statistical Methods Group (2019). “Analysing data and undertaking meta‐analyses,” in Cochrane handbook for systematic reviews of interventions. Editors Higgins J. P. T. Thomas J. Chandler J. Cumpston M. Li T. Page M. J. 1st ed. (Wiley), 241–284. 10.1002/9781119536604.ch10

[B15] Duval S. Tweedie R. (2000). Trim and fill: a simple funnel-plot–based method of testing and adjusting for publication bias in meta-analysis. Biometrics 56 (2), 455–463. 10.1111/j.0006-341x.2000.00455.x 10877304

[B16] Egger M. Smith G. D. Schneider M. Minder C. (1997). Bias in meta-analysis detected by a simple, graphical test. Bmj 315 (7109), 629–634. 10.1136/bmj.315.7109.629 9310563 PMC2127453

[B17] Elliott-Sale K. J. Minahan C. L. de Jonge X. A. K. J. Ackerman K. E. Sipilä S. Constantini N. W. (2021). Methodological considerations for studies in sport and exercise science with women as participants: a working guide for standards of practice for research on women. Sports Med. Auckl. N.Z. 51 (5), 843–861. 10.1007/s40279-021-01435-8 33725341 PMC8053180

[B18] Emmonds S. Heyward O. Jones B. (2019). The challenge of applying and undertaking research in female sport. Sports Med. - Open 5 (1), 51. 10.1186/s40798-019-0224-x 31832880 PMC6908527

[B19] Faude O. Koch T. Meyer T. (2012). Straight sprinting is the most frequent action in goal situations in professional football. J. Sports Sci. 30 (7), 625–631. 10.1080/02640414.2012.665940 22394328

[B20] Fischetti F. Cataldi S. Greco G. (2019). Lower-limb plyometric training improves vertical jump and agility abilities in adult female soccer players. 10.7752/jpes.2019.02182

[B21] Flemyng E. Moore T. H. Boutron I. Higgins J. P. Hróbjartsson A. Nejstgaard C. H. (2023). Using risk of bias 2 to assess results from randomised controlled trials: guidance from cochrane. BMJ Evidence-Based Med. 28 (4), 260–266. 10.1136/bmjebm-2022-112102 36693715

[B22] Gaamouri N. Hammami M. Cherni Y. Rosemann T. Knechtle B. Chelly M. S. (2023). The effects of 10-week plyometric training program on athletic performance in youth female handball players. Front. Sports Act. Living 5, 1193026. 10.3389/fspor.2023.1193026 37521098 PMC10375710

[B23] Guyatt G. Oxman A. D. Akl E. A. Kunz R. Vist G. Brozek J. (2011). GRADE guidelines: 1. Introduction-GRADE evidence profiles and summary of findings tables. J. Clin. Epidemiol. 64 (4), 383–394. 10.1016/j.jclinepi.2010.04.026 21195583

[B24] Haghighi A. H. Hosseini S. B. Askari R. Shahrabadi H. Ramirez-Campillo R. (2024). Effects of plyometric compared to high-intensity interval training on youth female basketball player’s athletic performance. Sport Sci. Health 20 (1), 211–220. 10.1007/s11332-023-01096-2

[B25] Hammami M. Ramirez-Campillo R. Gaamouri N. Aloui G. Shephard R. J. Chelly M. S. (2019). Effects of a combined upper- and lower-limb plyometric training program on high-intensity actions in female U14 handball players. Pediatr. Exerc. Sci. 31 (4), 465–472. 10.1123/pes.2018-0278 31310989

[B26] Hammami M. Gaamouri N. Suzuki K. Shephard R. Chelly M. (2020). Effects of upper and lower limb plyometric training program on components of physical performance in young female handball players. Front. PHYSIOLOGY 11, 1028. 10.3389/fphys.2020.01028 33013446 PMC7461999

[B27] Higgins J. P. T. Thompson S. G. (2002). Quantifying heterogeneity in a meta‐analysis. Statistics Med. 21 (11), 1539–1558. 10.1002/sim.1186 12111919

[B28] Hopkins W. Marshall S. Batterham A. Hanin J. (2009). Progressive statistics for studies in sports medicine and exercise science. Medicine+ Sci. Sports+ Exerc. 41 (1), 3–13. 10.1249/MSS.0b013e31818cb278 19092709

[B29] Hughes W. Healy R. Lyons M. Nevill A. Higginbotham C. Lane A. (2023). The effect of different strength training modalities on sprint performance in female team-sport athletes: a systematic review and meta-analysis. Sports Med. Auckl. N.Z. 53 (5), 993–1015. 10.1007/s40279-023-01820-5 36877405

[B30] Idrizovic K. Gjinovci B. Sekulic D. Uljevic O. Joao P. Spasic M. (2018). The effects of 3-month skill-based and plyometric Conditioning on fitness parameters in Junior female volleyball players. Pediatr. Exerc. Sci. 30 (3), 353–363. 10.1123/pes.2017-0178 29478378

[B31] Kellis S. E. Tsitskaris G. K. Nikopoulou M. D. Mousikou K. C. (1999). The evaluation of jumping ability of male and female basketball players according to their chronological age and major leagues. J. Strength and Cond. Res. 13 (1), 40–46. 10.1519/1533-4287(1999)013<0040:teojao>2.0.co;2

[B32] Komi P. V. (2003). Strength and power in sport. 1st ed. (Wiley). 10.1002/9780470757215

[B33] Kons R. L. Orssatto L. B. R. Ache-Dias J. De Pauw K. Meeusen R. Trajano G. S. (2023). Effects of plyometric training on physical performance: an Umbrella review. Sports Med. - Open 9 (1), 4. 10.1186/s40798-022-00550-8 36625965 PMC9832201

[B34] Kontopantelis E. Springate D. A. Reeves D. (2013). A re-analysis of the Cochrane Library data: the dangers of unobserved heterogeneity in meta-analyses. PloS One 8 (7), e69930. 10.1371/journal.pone.0069930 23922860 PMC3724681

[B35] Lambertz D. Mora I. Grosset J.-F. Pérot C. (2003). Evaluation of musculotendinous stiffness in prepubertal children and adults, taking into account muscle activity. J. Appl. Physiology 95 (1), 64–72. 10.1152/japplphysiol.00885.2002 12626487

[B36] Liberati A. Altman D. G. Tetzlaff J. Mulrow C. Gøtzsche P. C. Ioannidis J. P. A. (2009). The PRISMA statement for reporting systematic reviews and meta-analyses of studies that evaluate Health care interventions: explanation and elaboration. Ann. Intern. Med. 151 (4), W65–W94. 10.7326/0003-4819-151-4-200908180-00136 19622512

[B37] Lidor R. Ziv G. (2010). Physical characteristics and physiological attributes of adolescent volleyball players-a review. Pediatr. Exerc. Sci. 22 (1), 114–134. 10.1123/pes.22.1.114 20332545

[B38] Lloyd R. S. Meyers R. W. Oliver J. L. (2011). The natural development and trainability of plyometric ability during childhood. Strength and Cond. J. 33 (2), 23–32. 10.1519/SSC.0b013e3182093a27

[B39] Maciejczyk M. Błyszczuk R. Drwal A. Nowak B. Strzała M. (2021). Effects of short-term plyometric training on agility, jump and repeated sprint performance in female soccer players. Int. J. Environ. Res. Public Health 18 (5), 2274. 10.3390/ijerph18052274 33668937 PMC7956435

[B40] Maher C. G. Sherrington C. Herbert R. D. Moseley A. M. Elkins M. (2003). Reliability of the PEDro scale for rating quality of randomized controlled trials. Phys. Ther. 83 (8), 713–721. 10.1093/ptj/83.8.713 12882612

[B41] Malina R. M. Bouchard C. Bar-Or O. (2004). Growth, maturation, and physical activity. Champaign, IL: Human Kinetics.

[B42] Markovic G. Mikulic P. (2010). Neuro-Musculoskeletal and performance adaptations to lower-extremity plyometric training. Sports Med. 40 (10), 859–895. 10.2165/11318370-000000000-00000 20836583

[B43] Meszler B. Váczi M. (2019). Effects of short-term in-season plyometric training in adolescent female basketball players. Physiol. Int. 106 (2), 168–179. 10.1556/2060.106.2019.14 31271308

[B44] Miller M. G. Herniman J. J. Ricard M. D. Cheatham C. C. Michael T. J. (2006). The effects of a 6-week plyometric training program on agility. J. Sports Sci. and Med. 5 (3), 459–465. 24353464 PMC3842147

[B45] Moran J. J. Sandercock G. R. H. Ramírez-Campillo R. Meylan C. M. P. Collison J. A. Parry D. A. (2017). Age-related variation in Male youth athletes’ countermovement jump after plyometric training: a meta-analysis of controlled trials. J. Strength and Cond. Res. 31 (2), 552–565. 10.1519/JSC.0000000000001444 28129282

[B46] Moran J. Clark C. C. T. Ramirez-Campillo R. Davies M. J. Drury B. (2019). A meta-analysis of plyometric training in female youth: its efficacy and shortcomings in the literature. J. Strength and Cond. Res. 33 (7), 1996–2008. 10.1519/JSC.0000000000002768 30052601

[B47] Murr D. Raabe J. Höner O. (2018). The prognostic value of physiological and physical characteristics in youth soccer: a systematic review. Eur. J. Sport Sci. 18 (1), 62–74. 10.1080/17461391.2017.1386719 29161984

[B48] Nonnato A. Hulton A. T. Brownlee T. E. Beato M. (2022). The effect of a single session of plyometric training per week on fitness parameters in professional female soccer players: a randomized controlled trial. J. Strength and Cond. Res. 36 (4), 1046–1052. 10.1519/JSC.0000000000003591 32519832

[B49] Organization W. H. (2023). Global accelerated action for the Health of adolescents (AA-HA!): guidance to support country implementation. Geneva, Switzerland: World Health Organization.

[B50] Ortega-Becerra M. Belloso-Vergara A. Pareja-Blanco F. (2020). Physical and physiological demands during handball matches in male adolescent players. J. Hum. Kinet. 72 (1), 253–263. 10.2478/hukin-2019-0111 32269666 PMC7126253

[B51] Ozbar N. Ates S. Agopyan A. (2014). The effect of 8-week plyometric training on leg power, jump and sprint performance in female soccer players. J. Strength and Cond. Res. 28 (10), 2888–2894. 10.1519/JSC.0000000000000541 24852255

[B52] Paes P. P. Correia G. A. F. Damasceno V. O. Lucena E. V. R. Alexandre I. G. Da Silva L. R. (2022). Effect of plyometric training on sprint and change of direction speed in young basketball athletes. J. Phys. Educ. Sport 22 (2), 305–310. 10.7752/jpes.2022.02039

[B53] Page M. J. McKenzie J. E. Bossuyt P. M. Boutron I. Hoffmann T. C. Mulrow C. D. (2021). The PRISMA 2020 statement: an updated guideline for reporting systematic reviews. Bmj 372, n71. 10.1136/bmj.n71 33782057 PMC8005924

[B54] Pereira A. Costa A. M. Santos P. Figueiredo T. João P. V. (2015). Training strategy of explosive strength in young female volleyball players. Med. Kaunas. Lith. 51 (2), 126–131. 10.1016/j.medici.2015.03.004 25975882

[B55] Radnor J. M. Oliver J. L. Waugh C. M. Myer G. D. Moore I. S. Lloyd R. S. (2018). The influence of growth and maturation on stretch-shortening cycle function in youth. Sports Med. Auckl. N.Z. 48 (1), 57–71. 10.1007/s40279-017-0785-0 28900862 PMC5752749

[B56] Ramírez-Campillo R. Andrade D. C. Izquierdo M. (2013). Effects of plyometric training volume and training surface on explosive strength. J. Strength and Cond. Res. 27 (10), 2714–2722. 10.1519/JSC.0b013e318280c9e9 23254550

[B57] Ramírez-Campillo R. Vergara-Pedreros M. Henríquez-Olguín C. Martínez-Salazar C. Alvarez C. Nakamura F. Y. (2016). Effects of plyometric training on maximal-intensity exercise and endurance in Male and female soccer players. J. Sports Sci. 34 (8), 687–693. 10.1080/02640414.2015.1068439 26197721

[B58] Ramirez-Campillo R. García-Pinillos F. García-Ramos A. Yanci J. Gentil P. Chaabene H. (2018). Effects of different plyometric training frequencies on components of physical fitness in amateur female soccer players. Front. Physiology 9, 934. 10.3389/fphys.2018.00934 30065665 PMC6056896

[B59] Ramirez-Campillo R. Castillo D. Raya-González J. Moran J. De Villarreal E. S. Lloyd R. S. (2020a). Effects of plyometric jump training on jump and sprint performance in young male soccer players: a systematic review and meta-analysis. Sports Med. 50 (12), 2125–2143. 10.1007/s40279-020-01337-1 32915430

[B60] Ramirez-Campillo R. Sanchez-Sanchez J. Romero-Moraleda B. Yanci J. García-Hermoso A. Manuel Clemente F. (2020b). Effects of plyometric jump training in female soccer player’s vertical jump height: a systematic review with meta-analysis. J. Sports Sci. 38 (13), 1475–1487. 10.1080/02640414.2020.1745503 32255389

[B61] Ramirez-Campillo R. García-Hermoso A. Moran J. Chaabene H. Negra Y. Scanlan A. T. (2022). The effects of plyometric jump training on physical fitness attributes in basketball players: a meta-analysis. J. Sport Health Sci. 11 (6), 656–670. 10.1016/j.jshs.2020.12.005 33359798 PMC9729929

[B62] Rimmer E. Sleivert G. (2000). Effects of a plyometrics intervention program on sprint performance. J. Strength and Cond. Res. 14 (3), 295–301. 10.1519/00124278-200008000-00009

[B63] Rojano Ortega D. Berral-Aguilar A. J. Berral de la Rosa F. J. (2022). Kinetics and vertical stiffness of female volleyball players: effect of low-intensity plyometric training. Res. Q. Exerc. Sport 93 (4), 734–740. 10.1080/02701367.2021.1915946 34709134

[B64] Rosas F. Ramírez-Campillo R. Martínez C. Caniuqueo A. Cañas-Jamet R. McCrudden E. (2017). Effects of plyometric training and beta-alanine supplementation on maximal-intensity exercise and endurance in female soccer players. J. Hum. Kinet. 58, 99–109. 10.1515/hukin-2017-0072 28828081 PMC5548158

[B65] Sáez-Sáez de Villarreal E. Requena B. Newton R. U. (2010). Does plyometric training improve strength performance? A meta-analysis. J. Sci. Med. Sport 13 (5), 513–522. 10.1016/j.jsams.2009.08.005 19897415

[B66] Sánchez M. Sanchez-Sanchez J. Nakamura F. Y. Clemente F. M. Romero-Moraleda B. Ramirez-Campillo R. (2020). Effects of plyometric jump training in female soccer player’s physical fitness: a systematic review with meta-analysis. Int. J. Environ. Res. Public Health 17 (23), 8911. 10.3390/ijerph17238911 33266195 PMC7731275

[B67] Sánchez-Sixto A. Harrison A. J. Floría P. (2021). Effects of plyometric vs. Combined plyometric training on vertical jump biomechanics in female basketball players. J. Hum. Kinet. 77, 25–35. 10.2478/hukin-2021-0009 34168689 PMC8008314

[B68] Sedano Campo S. Vaeyens R. Philippaerts R. M. Redondo J. C. de Benito A. M. Cuadrado G. (2009). Effects of lower-limb plyometric training on body composition, explosive strength, and kicking speed in female soccer players. J. Strength and Cond. Res. 23 (6), 1714–1722. 10.1519/JSC.0b013e3181b3f537 19675492

[B69] Silva A. F. Ramirez-Campillo R. Ceylan H. İ. Sarmento H. Clemente F. M. (2022). Effects of maturation stage on sprinting speed adaptations to plyometric jump training in youth male team sports players: a systematic review and meta-analysis. Open Access J. Sports Med. 13, 41–54. 10.2147/OAJSM.S283662 35586483 PMC9109897

[B70] Stojanović E. Ristić V. McMaster D. T. Milanović Z. (2017). Effect of plyometric training on vertical jump performance in female athletes: a systematic review and meta-analysis. Sports Med. 47 (5), 975–986. 10.1007/s40279-016-0634-6 27704484

[B71] Stojanović E. Stojiljković N. Scanlan A. T. Dalbo V. J. Berkelmans D. M. Milanović Z. (2018). The activity demands and physiological responses encountered during basketball match-play: a systematic review. Sports Med. 48 (1), 111–135. 10.1007/s40279-017-0794-z 29039018

[B72] Vescovi J. D. Rupf R. Brown T. D. Marques M. C. (2011). Physical performance characteristics of high-level female soccer players 12–21 years of age. Scand. J. Med. and Sci. Sports 21 (5), 670–678. 10.1111/j.1600-0838.2009.01081.x 21917018

[B73] Wilk K. E. Voight M. L. Keirns M. A. Gambetta V. Andrews J. R. Dillman C. J. (1993). Stretch-shortening drills for the upper extremities: theory and clinical application. J. Orthop. Sports Phys. Ther. 17 (5), 225–239. 10.2519/jospt.1993.17.5.225 8343780

[B74] Young W. B. James R. Montgomery I. (2002). Is muscle power related to running speed with changes of direction? J. Sports Med. Phys. Fit. 42 (3), 282–288. 12094116

[B75] Zhang Y. Alonso-Coello P. Guyatt G. H. Yepes-Nuñez J. J. Akl E. A. Hazlewood G. (2019a). GRADE guidelines: 19. Assessing the certainty of evidence in the importance of outcomes or values and preferences-risk of bias and indirectness. J. Clin. Epidemiol. 111, 94–104. 10.1016/j.jclinepi.2018.01.013 29452223

[B76] Zhang Y. Coello P. A. Guyatt G. H. Yepes-Nuñez J. J. Akl E. A. Hazlewood G. (2019b). GRADE guidelines: 20. Assessing the certainty of evidence in the importance of outcomes or values and preferences-inconsistency, imprecision, and other domains. J. Clin. Epidemiol. 111, 83–93. 10.1016/j.jclinepi.2018.05.011 29800687

[B77] Zhou J.-Y. Wang X. Hao L. Ran X.-W. Wei W. (2024). Meta-analysis of the effect of plyometric training on the athletic performance of youth basketball players. Front. Physiology 15, 1427291. 10.3389/fphys.2024.1427291 39376898 PMC11457583

